# Barriers to the Utilization of Primary Health Centers (PHCs) in Iraq

**DOI:** 10.3390/epidemiologia4020013

**Published:** 2023-04-13

**Authors:** Taysir Al Janabi

**Affiliations:** New York Institute of Technology College of Osteopathic Medicine (NYITCOM), Glen Head, NY 11545, USA; taysir.aljanabi@gmail.com

**Keywords:** Iraq, primary care, primary health center, geographical inequality, health service utilization, patient satisfaction, referral system

## Abstract

Primary care has been viewed as a means to ensure equitable access to care, enhance efficiency within healthcare systems, and improve health service quality. In recent decades, Iraq has transformed its compromised health system, shifting the healthcare model from hospital-based to primary care through primary health centers (PHCs) and referral mechanisms. Based on an extensive literature review, this qualitative paper explores the healthcare utilization of PHCs in different regions of Iraq. It also identifies some barriers to PHC use and recommends evidence-based approaches for improving PHCs’ performance. Some reported challenges to better utilizing PHCs were the poor quality of services, patient dissatisfaction, long walking distance to a health center, and limited availability and affordability of the medications. If Iraq is to use primary care as a tool in achieving sustainable development goals (SDGs), collaborative efforts addressing the facility-related factors should be a priority.

## 1. Introduction

With aging citizens and rapid economic and population growth, the utilization of healthcare services has increased in Iraq, which necessitates an adjustment in the healthcare system’s structure and function to meet growing demands [1]. Equitable utilization of healthcare services is essential for individual and population wellbeing, as outlined by the United Nations’ initiative Sustainable Development Goals (SDGs) [2]. However, socioeconomic inequalities in healthcare utilization exist, and the situation is more prominent in low- and middle-income countries (LMICs) [3].

Low- and middle-income countries (LMICs) have an increased prevalence of multi-morbidity, which is closely associated with disability, premature deaths, unexpected hospitalizations, and high costs. Primary care has been considered critical in addressing this challenge [4]. However, in order to have high-performing primary care, the system should act as people’s first contact and offer patient-centered, comprehensive, and continuous care on a wide scale [5,6]; as such, many countries have begun transforming their healthcare systems with more emphasis on primary care.

With over four decades of war, conflicts, sanctions, and violence, Iraqis have been left with a deteriorating healthcare system and continuing environmental hazards, resulting in poor health outcomes [7]. The leading cause of death in Iraq is non-communicable diseases, including cardiovascular diseases, cancer, diabetes, and chronic lung diseases, which account for 62% of all deaths; this is lower compared to its neighboring countries, Jordan and Iran, where non-communicable diseases account for 76% and 77% of all deaths, respectively [8,9,10]. However, more than 20% of the non-communicable deaths among Iraqis occur in young individuals (the primary workforce)—compared to 20% in Jordan and 17% in Iran—resulting in negative economic and social impacts [11]. The Iraqi healthcare system has undergone a transformational change in recent decades, shifting health service delivery from curative and hospital-based to primarily preventive care-based through the use of primary health centers (PHCs) [12]. There are two kinds of PHCs: main and small subcenters. They are structurally different based on their urban or rural location [13]. All health services provided at the PHCs are offered at a discounted flat rate. However, certain health services for specific groups are free, such as preventive, maternal, newborn, and child health (MNCH). Individuals 60 years and older and others are also eligible for a fee waiver. To support the referral system, the referral hospital fees are waived if the referral occurred from a PHC to a referral hospital [14]. However, the inadequate distribution of health facilities, underfunding, physician shortages, and limited availability of medications have resulted in limited access to healthcare with significant geographical health disparities [15]; Iraq has 9.7 doctors and 23.8 nurses and midwives per 10,000 individuals, both of which are below the minimum recommended by the World Health Organization (WHO) [16,17,18]. Additionally, the Ministry of Health and Environment (hereafter called the Ministry of Health) operates on 1/12th of its regular budget [19].

The WHO has estimated that about 100 million individuals are pushed into poverty every year globally because of the out-of-pocket cost of health services [20]. Iraq has the lowest health expenditure per capita in the region with $154 in 2015, leading to poor health services and more financial burden imposed upon Iraqi citizens. It is estimated that an average out-of-pocket payment for an Iraqi person equals 70% of the health services they receive; the WHO has recommended that this ratio be less than 30% to protect patients from falling below the poverty line [11]. According to the 2021 Legatum Prosperity Index, the health pillar score of the country that assesses the extent to which people are healthy and have access to healthcare systems, the score increased slightly from 63.8 in 2011 to 65.5 in 2021; however, the country’s ranking moved down from 108 to 113 out of 167 countries. Additionally, Iraq ranked near the bottom in the Middle East and North Africa (MENA) region at 18 out of 19 countries [21].

Health service utilization is defined as the number of outpatient visits per person per year (public versus private) stratified by different clinic types, regions, age, and gender [22]. Health service utilization is reported as one of the indicators on the Global Reference List, developed by the WHO collaboratively with experts in the field; the list includes 100 core health indicators that can be monitored and evaluated to measure countries’ commitments toward the SDGs. Additionally, health service utilization also reflects the accessibility and functionality of health systems [22,23]. It is also viewed as the endpoint of health-seeking behavior [24]. The model by Levesque et al. views utilization as realized access, which can be easily measured. According to Levesque et al., five components related to health services influence the conceptual proposal of access: approachability; acceptability; availability and accommodation; affordability; and appropriateness. Additionally, five more person-related components influence access: the ability to perceive; the ability to seek; the ability to reach; the ability to pay; and the ability to engage [25], which are influenced by the social determinants of health [26].

This paper’s purpose is to describe the health service utilization in Iraq within the WHO’s definition of service utilization while focusing on the use of PHCs, highlighting the geographical variations in utilization, and discussing some of the elements that might be contributing to unequal access and utilization of health services. The paper also briefly assesses how the PHCs’ utilization aligns with the Levesque et al. model. The study may also offer areas of opportunity to address gaps and barriers to health service provisions.

## 2. Materials and Methods

### 2.1. Search Strategy

This paper is a qualitative analysis involving a comprehensive review of the academic literature, official government documents, and reports from credible national and international organizations. The search was performed in December 2022 and January 2023 and included studies published up to 21 January 2023. The literature search was conducted in the PubMed database, Cochrane Library, and Scopus database. The Cochrane database was searched for grey literature. A search was performed using the focused key medical subject heading (MeSH) terms for: “Socioeconomic inequalities in health”, “Geographical disparities in health”, “Utilization of inpatient/outpatient services”, and “Iraq”. The same search strategy from the PudMed database was applied to the Cochrane and Scopus databases. Results were imported into Endnote and deduplicated by the author. Additionally, the same keywords were applied to the Iraqi Academic Scientific Journals (IASJ) database to have a better understanding of the local context of health disparities and health service utilization. Moreover, a comprehensive review of the official website of the Government of Iraq was conducted to identify policies related to the health system. After deduplication, the search resulted in 334 papers: 74, 3, 199, and 58 from the PubMed database, Cochrane Library, Scopus database, and IASJ, respectively. Thirty-nine articles were included in the analysis. Of the 58 articles identified in the IASJ, 28 were in Arabic. Details of the search method are shown in the Supplementary File S1. Due to the nature of this study, approval by the Institutional Review Board was not required.

### 2.2. Data Extraction

An extensive desk review of the literature included the following inclusion and exclusion criteria: papers addressing health systems, health inequities, socioeconomic disparities in health, health priorities, patient acceptance of healthcare, healthcare facilities, manpower, inpatient and outpatient health service utilization, and the Iraqi context in general or one or more of its governorates, were included. Papers published before 2000 were excluded. In terms of sources, the author used reports and policy briefs from the United Nations (UN) and several of its agencies and missions in Iraq: WHO and the United Nations High Commissioner for Refugees (UNHCR). These documents provided a description of the health system and its strengths and weaknesses as well as specific health services provided to certain populations. The official websites of the Government of Iraq and its ministries (Ministry of Health and Ministry of Planning) were reviewed, and official documents were also examined for the latest assessments, plans, and statistics. The author also reviewed the relevant academic journals and international and national organizations’ reports—the World Bank, the United States Agency for International Development (USAID), KAPITA, and the Iraq Health Access Organization (IHAO)—as they provide a more in-depth assessment of the health profile of the country and the government and health sector responses to emerging health issues. Qualitative data were mainly extracted to align with the narrative nature of the study; however, quantitative data were incorporated to supplement the narrative synthesis and discussion.

## 3. Results

Iraq has 18 governorates, which can be grouped into five divisions consisting of groups of governorates with similar population sizes [27]. Table 1 shows the governorates and their demographics.

**Figure 1:**
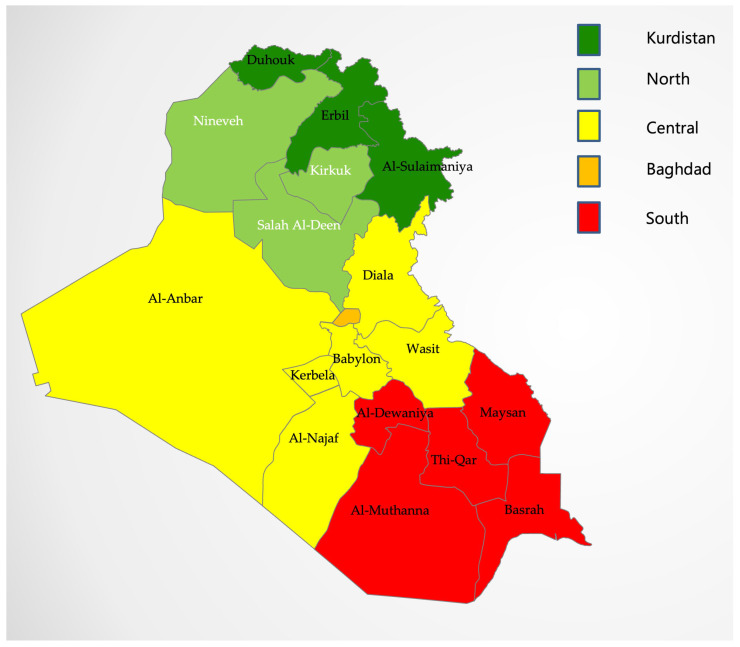
The Map of Iraq and its Governorates with its Five Divisions. Slide courtesy of SlideModel.com. https://slidemodel.com/templates/editable-iraq-powerpoint-map/ (accessed on 25 January 2023).

These divisions are Kurdistan (governorates of the Kurdistan Regional Government), North (three governorates directly south of Kurdistan and to the north of Baghdad), Baghdad (the single governorate of Baghdad, the capital city), Central (governorates to the east, west, and immediately south of Baghdad), and South (the five southern-most governorates of Iraq). Figure 1 shows the governorates and the five divisions.

### 3.1. Primary Health Centers (PHCs)

**Figure 2:**
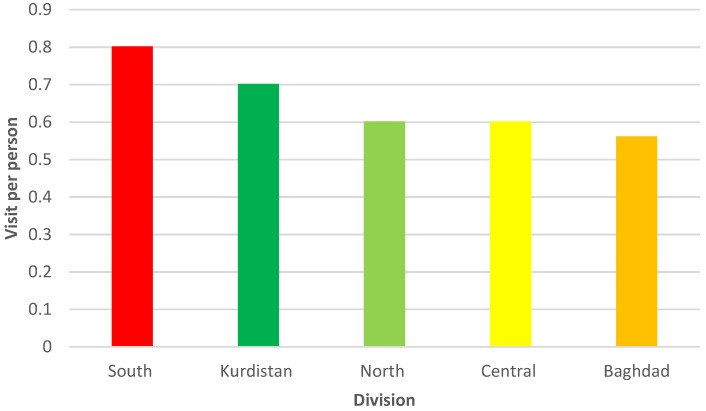
The Weighted per Capita Visit per Division of Iraq. Adopted from the annual statistical report 2020.

In 2009, Iraq defined the essential benefits package (EBP)—a package of health services that are funded publicly—and reviewed its definition in 2016 based on cost-effectiveness and budget impact, meaning identifying the interventions that maximize health benefits within the allocated budget. The Iraqi EBP is a comprehensive package linked to service provision in different health facilities, starting with community health and going up to the district hospital level [28]. PHCs can offer services covering preventive, promotive, and basic curative aspects of care. For example, PHCs can offer treatment for acute respiratory infections and refer the patient if the case is severe; measles with an eye infection or complications should be immediately referred to the district hospital [29]. The progressive decline in healthcare services, high staff turnover, and the inadequate distribution of health facilities and human resources have led citizens to bypass the PHCs and seek care at the secondary or tertiary level [11,12]. For example, Salah Al-Deen has 50% (842) of the needed doctors in the governorate available, while only 17 are located in rural regions [30]. Iraq has 1289 main PHCs, and each center serves 10,000–45,000 individuals, while it is projected that the country needs about 3000 main PHCs, and each one should serve about 10,000 people [11,18,31]. Additionally, according to planning standards in Iraq, a PHC should be within 700 m of an individual’s residence, and citizens should be able to get to the nearest PHC within 10 min by walking [31]. The USAID’s Primary Health Care Project in Iraq (PHCPI) reported that the most utilized services at PHCs were maternal and child health, immunization, and school health, while nutrition and health education were not adequately delivered. Moreover, the majority of the PHC users were there for therapeutic reasons because they could afford the prescribed medications, yet only a small portion of the prescribed medicines were available. It was also reported that men and women were treated unequally at PHCs [32]. Figure 2 shows the weighted per capita visit according to each division.

A referral system is essential for primary care as it serves as a means to coordinate clinical care between different facilities, streamline the continuity of patient care, and improve the quality of care [33]. Eighty-five percent of PHCs in Iraq have a referral system, yet 64% of these centers reported a lack of a feedback mechanism about these referrals [34]. A study that explored the effect of implementing a referral system in the governorate of Wasit showed that after three months of applying the referral system, the number of visits to the local hospital was reduced by one-third compared to the period prior, and the number of PHC visits doubled for the same period [35].

Childhood immunization was reported as one of the most common reasons to use PHCs; however, a lack of knowledge about the need for vaccinations and their timing, along with a lack of information about where the vaccines are available were reported as the main barrier to not getting vaccinated or not completing the vaccination for children in Iraq [36]. Additionally, low literacy is associated with a lack of medical information, less use of preventive care services, lower medication adherence rates, high rate of hospitalization, and higher health care cost [37,38]. The literacy rate among Iraqi adults is only 43.7% [39]. Moreover, self-medication is a common practice in Iraq; thus, the public can access a wide range of medicines. Most people who self-medicate get their medications from pharmacies, street vendors, or their families before consulting with a medical professional [40]. Lastly, Iraq has no regulatory policy for registering medicines as prescription-only or over-the-counter medications [40].

In general, there is a high preference for using private physicians to provide primary care services, 53–60%, compared to 16–21% provided by the public sector [41]. The wide availability of private sector health services and easy accessibility has increased private sector utilization for those who can afford its cost. Additionally, the increased number of specialist physicians in Iraq combined with the inability of the hospitals to absorb that number has resulted in increased private practice [41].

### 3.2. Results by Region

#### 3.2.1. Kurdistan

In Duhouk, PHC visits accounted for 65% of total outpatient visits in 2020, while Al-Sulaimaniya reported that only 27% of visits were PHC-related. PHC visits in Erbil accounted for 50% of the total outpatient visits [18]. All the PHCs provide the core primary care services to a variable extent. For example, PHCs in Al-Sulaimaniya provide the highest level of service coverage with 75–100%, while Duhouk offers the lowest level of service with 49–71%. However, Duhouk is the only governorate that offers mental health screening and management services to a large extent because of its many refugees and displaced individuals [42]. Most PHCs in Kurdistan have a referral system; however, there is no method of receiving the information back from referral hospitals. Additionally, the PHCs’ subcenters do not have a referral system [43].

#### 3.2.2. North

PHC patient visits in 2020 were reported to be 67%, 59%, and 47% of the total outpatient visits in Nineveh, Kirkuk, and Salah Al-Deen, respectively; females contributed to 54% of these visits [18]. The majority of the PHC attendants in Nineveh reported that they were satisfied with the essential health services offered by PHCs, and their satisfaction was significantly associated with the six domains of care (accessibility, acceptability, confidentiality, thoroughness, informativeness, and continuity) [44]. Additionally, Iraqis in Nineveh prefer to receive vaccines at PHCs instead of private clinics because public health facilities offer free vaccines and people trust the vaccines imported by the government. A lack of education and limited health facilities were also reported as barriers to vaccination.

In the Salah Al-Deen governorate, people were not satisfied with the health services provided by the PHCs, and the main reason cited was that PHCs did not provide essential health services; there was a general agreement that the services offered by the public sector were inferior to those provided by the private sector [45].

#### 3.2.3. Baghdad

PHC patient visits in 2020 were reported to be 46% of the total outpatient visits, comparable to hospital outpatient visits; females were responsible for about 55% of the visits [18]. The available literature suggests that there is an adequate level of quality of care offered by urban and rural PHCs in Baghdad; however, some challenges have been reported, such as unnecessary visits, high workload, long waiting times, and a shortage of medicines, which have been attributed to organizational and operational issues [46,47].

#### 3.2.4. Central

The total PHC patient visits in 2020 constituted 50% of the total outpatient visits, with the highest being 66% in Diala and the lowest being 44% in Babylon; females contributed to 56% of the total visits [18]. After the Islamic State in Iraq and Syria (ISIS) occupation of Nineveh, about 190,000 internally displaced persons (IDPs) moved to Diala. The sudden and rapid increase in IDPs added an additional burden on the available preventive and curative health services because of the lack of medical supplies, limited human resources, and inadequate basic services [48].

A study in the governorate of Al-Anbar explored patients’ perception of the quality of health services, which was reported to be of poor quality by 93% of PHC attendants. Additionally, participants reported that about 64% walked more than 15 min to the nearest PHC [49]. In Babylon, a study that explored the quality of healthcare services provided at a primary care center found that the health professionals’ attitude was a determinant of patients’ satisfaction with the center [50].

#### 3.2.5. South

The total PHC patient visits in 2020 constituted 48% of the total outpatient visits, with the highest being 55% in Thi-Qar and the lowest being 38% in Maysan; female visits constituted about 56% [18]. The most important factors affecting utilization in the South were the level of perceived sickness in the family and the distance to the nearest health center; the cost of the visit was not a factor in health service utilization at PHCs except when the private sector provides care [26]. Patient dissatisfaction was reported as a barrier to health utilization at PHCs in at least two governorates, Thi-Qar and Basrah, and other regions within the divisions [51,52,53].

The qualitative and quantitative defects in health services have led people from rural areas to seek the healthcare they need in urban regions. For example, the city of Warkaa in Al-Muthanna has 3684 individuals, and only one PHC provides essential health services; this PHC provided services to a total population of 51,000 because of the rural residents seeking care in the cities due to the lack of PHCs in their areas. The majority of residents spend more than 20 min walking to the nearest PHC with an average of 27 and 60 min in urban and rural areas, respectively [31]. Patient satisfaction with their healthcare was reported to be 42.9% and 44.5% in rural and urban settings, respectively [54].

### 3.3. Vulnerable Groups

#### 3.3.1. Age-Friendly PHCs

The Ministry of Health has provided training about geriatric health services to specific PHC staffs designated as age-friendly centers; these PHCs aim to improve life expectancy and quality of life. There are currently 57 age-friendly centers, with 31 of them in Baghdad [15]. While health services for the elderly in the PHCs are free, twenty-two of the age-friendly centers in Baghdad did not offer free services. Less than 25% of these clinics have a suitable water source, most lack a referral system, and most health staff lack adequate training in geriatric health services. The cost associated with health visits is reported to be one of the reasons for the underutilization of health services by geriatrics; about one older adult per day attends one of these PHCs [55].

#### 3.3.2. Urban–Rural Gap

Health coverage inequalities based on residence in Iraq exist; a study observed that urban–rural health coverage inequalities have decreased since 2000; however, inequalities will still exist in 2030, mainly for the third dose of diphtheria, tetanus toxoid, and pertussis (DTP3); complete immunization; measles; and antenatal care visits one and four. Iraqis spend on average 6.5% of their total household income on health services; the highest was among the residents in Basrah and Al-Najaf, who spend more than 10% of their income, then residents of Kurdistan and Baghdad, who spend 7–9% of their income. The lowest expenditure was among the residents of Al-Muthanna with 1.8% [56]. Additionally, rural residents, the elderly, female-led households, and households with children under five years old are at an increased risk of catastrophic health expenditures. The urban–rural gap in health expenditure was more pronounced in Erbil and Duhouk in Kurdistan, Salah Al-Deen in the North, Wasit in the Central, and Al-Muthanna in the South [57].

#### 3.3.3. Antenatal Care (ANC)

The current literature has shown that the quality of ANC at ten PHCs in Babylon was associated with the availability of medicines, the ability to discuss pregnancy-related issues, and the privacy level provided at the PHC. Additionally, most respondents (83%) reported being satisfied with the services provided [58].

Although antenatal care is reported to be affordable at PHCs, twenty percent of pregnant women in rural areas preferred the private sector. Barriers to antenatal care at PHCs in rural Basrah were the harsh attitude of healthcare workers at these centers, lack of medical supplies and medicines, and longer waiting times. Additionally, difficulty watching older children at home was another barrier. Moreover, cultural attitudes, distance to PHCs, and transportation were also reported as challenges to ANC utilization [59].

### 3.4. Health Utilization as a Realized Access

In Iraq, the limited available literature suggests that health services are approachable; however, limitations exist regarding their acceptability (rural vs. urban), availability (inequitable distribution of health facilities and human resources), affordability (public vs. private), and appropriateness (lack of health services for the expressed need, longer waiting times, and inadequate referrals and continuity of care) [60]. Table 2 outlines some of the strengths, weaknesses, opportunities, and threats (SWOT) of Iraqi PHCs in general.

## 4. Discussion

According to our paper, the coverage of essential health services was below the international standards of 90–100% set by the WHO [61]. The utilization of primary health services at PHCs varied across the five divisions and even within each division. The North division had the highest rate of PHC visits at 58% in the country; Nineveh had the highest rate of PHC visits compared to Salah Al-Deen, which had the lowest in the division; the same pattern can be recognized in Kurdistan, where the highest PHC utilization was in Duhouk and significantly low in Al-Sulaimaniya, which has the highest number of PHCs with the highest service coverage. This variation might be due to the perception of the quality of health services at PHCs. Additionally, the high number of people in the region (refugees and IDPs) might have contributed to this high utilization where they were resettled [62].

Health utilization and perception of the quality of health services are interrelated as the quality perception can influence health service utilization [41]. Additionally, health service utilization is affected by accessibility, affordability, health needs, resources, and socioeconomic and cultural factors [41,63]. Patient satisfaction and experiences with health services have been used as a measure of service quality, which has been determined to be a critical factor in health service utilization [64,65]. Our study reports that patient dissatisfaction could be a factor in not seeking care from PHCs, a finding consistent with the available literature [66,67]. A study that explored the utilization of primary care services in Ibadan, Nigeria observed that only satisfaction with care received was a predictor of health service utilization in the area [68]. Additionally, there is a general inclination toward the private sector, for those who can afford the cost, which was attributed to better services and provider–patient interactions. Health professional attitudes toward people utilizing health services at public facilities were a determinant of patient satisfaction in Bangladesh [69]. The current literature has documented that patient satisfaction leads to improved compliance, increased health service utilization, and better health outcomes [44]. 

Some of the contributing factors to patients’ poor experiences are inadequate training of health professionals regarding physician–patient interactions, high workload, perceived threats by health professionals, and lack of predictable patient visits [70]. In the context of Iraq, the inclusion of provider–patient communication skills in the medical education curricula could help improve the patient experience since the Iraqi rural population has a culture with unique beliefs and practices about health; they view health and disease differently from the physicians, impacting doctor–patient communication [71]. A study that explored the use of PHCs for communicable diseases in Southeast Nigeria showed that better provider–patient interactions resulted in increased use of PHCs [72]. Additionally, minimizing the high workload and establishing an appointment system might improve the patient experience. Patients can visit any PHC of their preference and be served on a walk-in basis, which offers easy access to the health system; however, it also imposes the challenge of repeated unnecessary visits and workload, resulting in poor provider–patient interactions, longer waiting times, and severe medicine shortages. Thus, introducing a well-organized appointment system could increase the utilization of expensive human and medical resources and organize the work of PHCs [47]. Moreover, adequate distribution of human resources could mitigate the high work demand.

The distance of a PHC from an individual’s residence was a factor in the utilization of PHCs in several governorates, mainly in the South. Our finding was consistent with the current literature. A study exploring the encouraging factors for PHC utilization in Al-Madinah, Saudi Arabia observed that the close proximity of PHCs to the user’s residence was the main reason for seeking care at PHCs [67]. Another study in India observed that the main reason to seek care from a specific health facility was reported to be the accessibility of that facility [60]. Iraq still needs new PHCs, which could be an opportunity to mitigate the impact of distance by equitably distributing these centers based on population growth and health needs.

Childhood vaccination was one of the reasons for using PHCs. Children who initiate their vaccination at a public facility are more likely to complete their vaccination schedule; however, the unavailability of the vaccines at these sites was reported as a barrier to health service utilization [73,74]. Thus, the availability of vaccines and vaccine-associated services—documentation and health education—might result in the constant use of PHCs. Additionally, the PHCs could be used as vaccination sites for future health emergencies in the same way they were used in Cuba, where adequately equipped and well-distributed primary health centers served as vaccination sites for COVID-19 vaccines [75].

The affordability and availability of the medications prescribed at the PHCs were reported as another reason to utilize PHCs; however, not all essential medications are always available. A comprehensive review conducted by the Ministry of Health to assess the percentage of the essential medications (531 items) that were available in 2018 showed that only 12% of the medications list was completely available throughout the year, 39% of the list was available inconsistently, and about 49% was completely unavailable all year long [11]. A cross-sectional study in Enugu, Nigeria identified that the unavailability of medicines was a reason for not seeking care at a local PHC. Medication availability was an important determinant of patient satisfaction [76]. Making the essential medications consistently available might attract new and retain current PHC users and create opportunities for future utilization of these centers. 

In addition to the measures mentioned above for optimizing PHCs’ performance, more financial resources should be allocated to PHCs to fund some activities, such as health education and promotion, to address non-communicable diseases. Health policies at the national level should set the minimum standard of care at PHCs and launch PHC accreditation programs [77,78]. Establishing and reinforcing a referral system is essential as it has advantages such as avoiding the wasteful use of limited resources, streamlining the continuity of care, and improving the quality of care [79,80]. Public–private partnerships to support PHCs should also be considered. Such partnerships have been shown to expand access, increase service coverage, and improve the quality of health services, especially in remote areas [81,82]. Personal and professional incentives should be considered to encourage physicians to enter family medicine training programs; Iraq has about 500 family physicians, while the estimated need is in the thousands [11]. Moreover, incentives should be offered to the public for using the referral system, such as fee waivers, discounts at the local referral hospital, or receiving priority when seeking care at the local hospital.

### Limitations

The paper is qualitative in nature and is based on a search of the published literature. Thus, the study is limited by the number of databases used, as the author might have missed some of the literature outside these databases. Additionally, this paper’s findings can neither be used for generalization nor causation. Moreover, the lack of uniformity of the reviewed studies limited a comprehensive overview of healthcare utilization nationwide; however, this paper identified common themes affecting healthcare utilization at PHCs. Lastly, the effects of time (given the range of years) and the fact that only one author was synthesizing information could also be limitations.

## 5. Conclusions

Primary health care is a core element for achieving equity and efficiency within a healthcare system, and it is an essential tool for achieving universal health coverage (UHC) in 2030. Thus, restructuring and modernizing PHCs is critical to improving the deteriorating healthcare system. To do so, a top priority should be given to collaborative efforts addressing the facility-related factors influencing PHC utilization in Iraq.

## Figures and Tables

**Table 1:** Iraq’s Governorates and their Demographics.

Governorate	Population	Females	Male	Urban (%)	Rural (%)	PHC Number	Per Capita Visit
Baghdad	8,558,625	4,215,859	4,342,766	87.5	12.5	260	0.56
Basrah	3,063,059	1,524,286	1,538,773	81.2	18.8	135	0.90
Nineveh	3,928,215	1,921,564	2,006,651	60.6	39.4	185	0.67
Maysan	1,171,802	588,036	583,766	73.9	26.1	79	0.43
Al-Dewaniya	1,359,642	674,362	685,280	57.3	42.7	84	0.51
Diala	1,724,238	853,239	870,999	49.2	50.8	102	0.81
Al-Anbar	1,865,818	907,275	958,543	50.0	50.0	187	0.43
Babylon	2,174,783	1,075,899	1,098,884	48.3	51.7	121	0.59
Kerbela	1,283,484	636,022	647,462	66.9	33.1	62	0.88
Kirkuk	1,682,809	835,689	847,120	73.9	26.1	127	0.38
Wasit	1,452,007	718,986	733,021	60.2	39.8	78	0.69
Thi-Qar	2,206,514	1,098,993	1,107,521	64.2	35.8	168	0.64
Al-Muthanna	857,652	426,675	430,977	46.4	53.6	71	0.70
Salah Al-Deen	1,680,015	831,380	848,635	45.1	54.9	129	0.50
Al-Najaf	1,549,788	772,754	777,034	71.4	28.6	82	0.74
Erbil	1,953,341	967,192	986,149	83.2	16.8	272	0.57
Duhouk	1,361,211	679,122	682,089	74.1	25.9	171	1.14
Al-Sulaimaniya	2,277,171	1,138,018	1,139,153	84.7	15.3	492	0.54

Adopted from the annual statistical report 2020.

**Table 2:** The SWOT Table of Iraqi PHCs.

	Strengths	Weaknesses
	Resilience of the health system Approachability Private sector	Inequitable distribution of health facilities and human resources Lack of national regulatory health policies Lack of adequate funding Lack of adequate referral system
**Opportunities**	Increase and sustain the availability of childhood vaccinations Increase and sustain the availability of essential medications Leverage health education in communicable and non-communicable disease awareness campaigns	Strategic planning for adequate distribution based on population size and health needs Establish and distribute mobile clinics for primary health services Explore self-funding and private funding opportunities
Childhood immunization at PHCs Affordability of health services and medications Health education		
**Threats**	Build and maintain public–private partnerships Encourage private sector investment in PHCs Offer incentives for individuals and health professionals	Increase the use of hospital-based health services Decrease in the PHCs’ service coverage Decline in individuals’ health status
Low service coverage by PHCs Patient dissatisfaction		

## Data Availability

Not applicable.

## Supplementary Materials

The following supporting information can be downloaded at: https://www.mdpi.com/article/10.3390/epidemiologia4020013/s1, Supplementary File S1: Literature search methodology.
