# Supplementary material for: Barriers to the Utilization of Primary Health Centers (PHCs) in Iraq
Source: Epidemiologia (Basel). 2023 Apr 13;4(2):121–33. doi: 10.3390/epidemiologia4020013 (PMC10123605; doi:10.3390/epidemiologia4020013)
Supplement: Supplementary file 1 [file epidemiologia-04-00013-s001.zip › epidemiologia-2307664-supplementary.pdf]

**Research Question**	**Databases, Resources, and Limits**	
The socioeconomic inequalities in health: geographical disparities and utilization of inpatient/outpatient services in Iraq	**Select Core Databases:**<br>PubMed<br>Cochrane Library<br>Scopus<br>Iraqi Academic Scientific Journals (IASJ)	**Limits:**

Database: PubMed

	**Concept: Socioeconomic inequalities in health**	**Concept: Geographical disparities in health**	**Concept: Utilization of inpatient/outpatient services**	**Concept: Iraq**
**Thesaurus Terms/ Subheadings**				
**Textwords**	“Socioeconomic inequalities in health” OR “Health Inequities” OR “Socioeconomic Factors” OR “Socioeconomic Disparities in Health” OR “Healthcare Disparities” OR “Low Socioeconomic Status” OR “Health Priorities” OR “Patient Acceptance of Health Care”	“Geographical disparities in health” OR “Health Status Disparities” OR “Health Disparate, Minority and Vulnerable Populations” OR “Healthcare Disparities” OR “Health Services Accessibility” OR “Global Health” OR “Health Care Facilities, Manpower, and Services”	“Utilization of inpatient outpatient health services” OR “Ambulatory Care” OR “Facilities and Services Utilization” OR “Health Services Misuse” OR “Health Services” OR “Rural Health Services” OR “Utilization Review” OR “Drug Utilization”	“Iraq” Or<br>Republic of Iraq

Line 1: "socioeconomic factors"[MeSH Terms] OR ("socioeconomic"[All Fields] AND "factors"[All Fields]) OR "socioeconomic factors"[All Fields] OR "socioeconomics"[All Fields] OR "socioeconomic"[All Fields] OR "socioeconomical"[All Fields] OR "socioeconomically"[All Fields]) AND ("inequalities"[All Fields] OR "inequality"[All Fields] OR "inequities"[All Fields] OR "inequity"[All Fields])) AND "health"[MeSH Terms]) OR "health inequities"[MeSH Terms] OR "socioeconomic factors"[MeSH Terms] OR (("socioeconomic disparities in health"[MeSH Terms] OR ("socioeconomic"[All Fields] AND "disparities"[All

Fields] AND "health"[All Fields]) OR "socioeconomic disparities in health"[All Fields] OR ("socioeconomic"[All Fields] AND "disparities"[All Fields]) OR "socioeconomic disparities"[All Fields]) AND "health"[MeSH Terms]) OR "healthcare disparities"[MeSH Terms] OR "low socioeconomic status"[MeSH Terms] OR "health priorities"[MeSH Terms] OR "patient acceptance of health care"[MeSH Terms]
Results: 692,754

Line 2: "Health Status Disparities"[MeSH Terms] OR "health disparate, minority and vulnerable populations"[MeSH Terms] OR "Healthcare Disparities"[MeSH Terms] OR "Health Services Accessibility"[MeSH Terms] OR "Global Health"[MeSH Terms] OR "health care facilities, manpower, and services"[MeSH Terms]
Results: 3,502,870

Line 3: Line 1 + Line 2
Results: 328,662

Line 4: "statistics and numerical data"[MeSH Subheading] OR ("statistics"[All Fields] AND "numerical"[All Fields] AND "data"[All Fields]) OR "statistics and numerical data"[All Fields] OR "utilization"[All Fields] OR "utilisation"[All Fields] OR "utilisations"[All Fields] OR "utilise"[All Fields] OR "utilised"[All Fields] OR "utilises"[All Fields] OR "utilising"[All Fields] OR "utilities"[All Fields] OR "utility"[All Fields] OR "utilizations"[All Fields] OR "utilize"[All Fields] OR "utilized"[All Fields] OR "utilizer"[All Fields] OR "utilizers"[All Fields] OR "utilizes"[All Fields] OR "utilizing"[All Fields]) AND ("inpatient s"[All Fields] OR "inpatients"[MeSH Terms] OR "inpatients"[All Fields] OR "inpatient"[All Fields])) AND "ambulatory care"[MeSH Terms]) OR "ambulatory care"[MeSH Terms] OR "facilities and services utilization"[MeSH Terms] OR "health services misuse"[MeSH Terms] OR "health services"[MeSH Terms] OR "rural health services"[MeSH Terms] OR "utilization review"[MeSH Terms] OR "drug utilization"[MeSH Terms]
Results: 2,421,119

Line 5: Line 3 + Line 4
Results: 211,131

Line 6: "iraq"[MeSH Terms]
Results: 5,357

Line 7: Line 5 + Line 6
Results: 74

Date: 01/20/2023

Database: Cochrane Library

	Concept: Socioeconomic inequalities in health	Concept: Geographical disparities in health	Concept: Utilization of inpatient/outpatient services	Concept: Iraq
**Thesaurus Terms/ Subheadings**				
**Textwords**	"Socioeconomic inequalities in health" OR "Health Inequities" OR "Socioeconomic Factors" OR "Socioeconomic Disparities in Health" OR "Healthcare Disparities" OR "Low Socioeconomic Status" OR "Health Priorities" OR "Patient Acceptance of Health Care"	"Geographical disparities in health" OR "Health Status Disparities" OR "Health Disparate, Minority and Vulnerable Populations" OR "Healthcare Disparities" OR "Health Services Accessibility" OR "Global Health" OR "Health Care Facilities, Manpower, and Services"	"Utilization of inpatient outpatient health services" OR "Ambulatory Care" OR "Facilities and Services Utilization" OR "Health Services Misuse" OR "Health Services" OR "Rural Health Services" OR "Utilization Review" OR "Drug Utilization"	"Iraq" Or Republic of Iraq

Line 1: "Socioeconomic inequalities in health" OR "Health Inequities" OR "Socioeconomic Factors" OR "Socioeconomic Disparities in Health" OR "Healthcare Disparities" OR "Low Socioeconomic Status" OR "Health Priorities" OR "Patient Acceptance of Health Care"
Results: 8,078

Line 2: "Geographical disparities in health" OR "Health Status Disparities" OR "Health Disparate, Minority and Vulnerable Populations" OR "Healthcare Disparities" OR "Health Services Accessibility" OR "Global Health" OR "Health Care Facilities, Manpower, and Services"
Results: 6,926

Line 3: Line 1 + Line 2
Results: 603

Line 4: "Utilization of inpatient outpatient health services" OR "Ambulatory Care" OR "Facilities and Services Utilization" OR "Health Services Misuse" OR "Health Services" OR "Rural Health Services" OR "Utilization Review" OR "Drug Utilization"
Results: 26,049

Line 5: Line 3 + Line 4
Results: 319

Line 6: "Iraq" Or Republic of Iraq
Results: 1,219

Line 7: Line 5 + Line 6
Results: 3

Date: 01/20/2023

Database: Scopus

	**Concept: Socioeconomic inequalities in health**	**Concept: Geographical disparities in health**	**Concept: Utilization of inpatient/outpatient services**	**Concept: Iraq**
**Thesaurus Terms/ Subheadings**				
**Textwords**	"Socioeconomic inequalities in health" OR "Health Inequities" OR "Socioeconomic Factors" OR "Socioeconomic Disparities in Health" OR "Healthcare Disparities" OR "Low Socioeconomic Status" OR "Health Priorities" OR "Patient Acceptance of Health Care"	"Geographical disparities in health" OR "Health Status Disparities" OR "Health Disparate, Minority and Vulnerable Populations" OR "Healthcare Disparities" OR "Health Services Accessibility" OR "Global Health" OR "Health Care Facilities, Manpower, and Services"	"Utilization of inpatient outpatient health services" OR "Ambulatory Care" OR "Facilities and Services Utilization" OR "Health Services Misuse" OR "Health Services" OR "Rural Health Services" OR "Utilization Review" OR "Drug Utilization"	"Iraq" Or Republic of Iraq

Line 1: "Socioeconomic inequalities in health" OR "Health Inequities" OR "Socioeconomic Factors" OR "Socioeconomic Disparities in Health" OR "Healthcare Disparities" OR "Low Socioeconomic Status" OR "Health Priorities" OR "Patient Acceptance of Health Care"
Results: 255,305

Line 2: “Geographical disparities in health” OR “Health Status Disparities” OR “Health Disparate, Minority and Vulnerable Populations” OR “Healthcare Disparities” OR “Health Services Accessibility” OR “Global Health” OR “Health Care Facilities, Manpower, and Services”
Results: 172,095

Line 3: Line 1 + Line 2
Results: 41,459

Line 4: “Utilization of inpatient outpatient health services” OR “Ambulatory Care” OR “Facilities and Services Utilization” OR “Health Services Misuse” OR “Health Services” OR “Rural Health Services” OR “Utilization Review” OR “Drug Utilization”
Results: 1,792,559

Line 5: Line 3 + Line 4
Results: 27,843

Line 6: “Iraq” Or Republic of Iraq
Results: 262,459

Line 7: Line 5 + Line 6
Results: 199

Date: 01/20/2023

Database: **Iraqi Academic Scientific Journals (IASJ)**

	**Concept: Socioeconomic inequalities in health**	**Concept: Geographical disparities in health**	**Concept: Utilization of inpatient/outpatient services**	**Concept: Iraq**
**Thesaurus Terms/ Subheadings**				
**Textwords**	“Socioeconomic inequalities in health” OR “Health Inequities” OR	“Geographical disparities in health” OR “Health Status Disparities” OR	“Utilization of inpatient outpatient health services” OR “Ambulatory	“Iraq” Or Republic of Iraq

	"Socioeconomic Factors" OR "Socioeconomic Disparities in Health" OR "Healthcare Disparities" OR "Low Socioeconomic Status" OR "Health Priorities" OR "Patient Acceptance of Health Care"	"Health Disparate, Minority and Vulnerable Populations" OR "Healthcare Disparities" OR "Health Services Accessibility" OR "Global Health" OR "Health Care Facilities, Manpower, and Services"	Care" OR "Facilities and Services Utilization" OR "Health Services Misuse" OR "Health Services" OR "Rural Health Services" OR "Utilization Review" OR "Drug Utilization"	

Each textword was used to perform a literature search, which resulted in 58 relevant articles.

Date: 01/21/2023
